# Endoscopic third ventriculostomy for hydrocephalus accompanied by dural arteriovenous fistulae: a case report and literature review

**DOI:** 10.1007/s00701-024-06418-y

**Published:** 2025-01-11

**Authors:** Daisuke Wajima, Tomoya Kamide, Yasuo Sasagawa, Sho Takata, Kouichi Misaki, Mitsutoshi Nakada

**Affiliations:** Department of Neurosurgery, Kanazawa University, 13-1 Takara-machi, Kanazawa, 9208641 Ishikawa Japan

**Keywords:** Endoscopic third ventriculostomy, Hydrocephalus, Thrombosis, Dural arteriovenous fistulae

## Abstract

A 54-year-old man presented with gait disturbances, urinary incontinence, and headache for 6 months. Head computed tomography indicated several high-density mass lesions in the quadrigeminal cistern, causing occlusive hydrocephalus. Digital subtraction angiography confirmed tentorial dural arteriovenous fistulae (AVF). Transarterial embolization (TAE) achieved complete angiographic resolution. However, acute occlusive hydrocephalus worsened, necessitating endoscopic third ventriculostomy (ETV). The patient was discharged without new symptoms and no hydrocephalus recurrence at six-month follow-up. Hydrocephalus is rare in patients with dural AVF and mostly resolves spontaneously after treatment; however, if thrombosis and enlargement of the varix occur after treatment, acute occlusive hydrocephalus can develop.

## Introduction

 Dural arteriovenous fistula (dural-AVF) is a vascular lesion associated with direct shunts between the dural arteries and cerebral venous sinuses and/or cortical veins. Symptoms depend on the site and pattern of venous drainage and can lead to cerebral hemorrhage or venous infarction. However, only seven cases of dural AVFs linked to obstructive hydrocephalus have been reported [3, 8–12, 14, 15]. Here, we present a case of falcotentorial dural AVF linked to obstructive hydrocephalus treated with transarterial embolization (TAE) and endoscopic third ventriculostomy (ETV).

## Case presentation

**Fig. 1:**
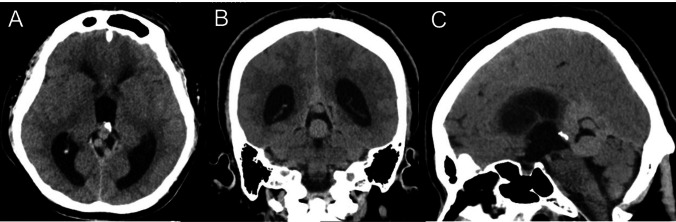
Initial head computed tomography (CT) of the patient, revealed several relatively high-density mass lesions in quadrigeminal cistern, resulting in occlusive hydrocephalus (**A**: axial view, **B**: coronal view, **C**: sagittal view)

**Fig. 2:**
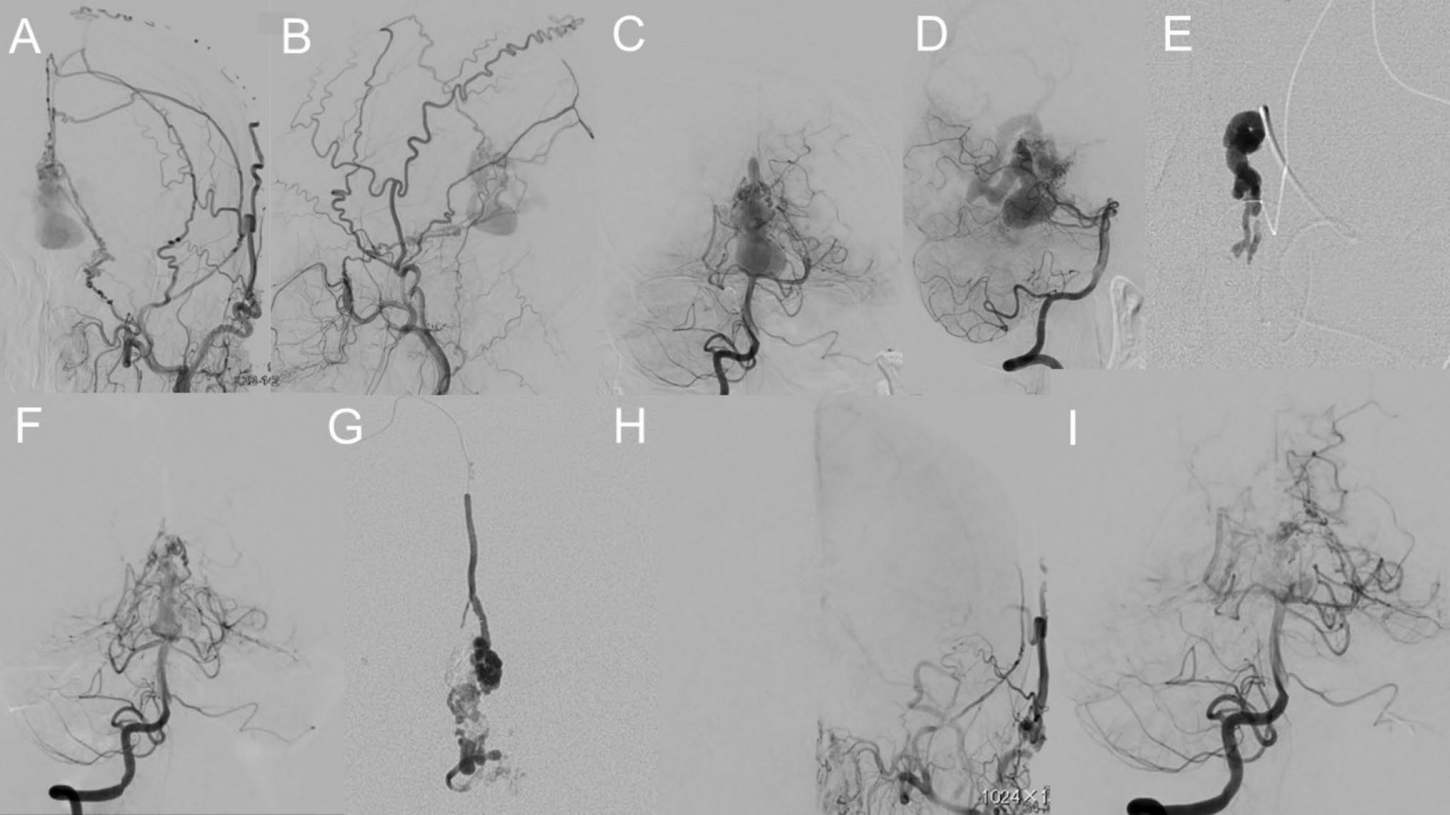
Digital subtraction angiography (DSA) revealed a tentorial dural AVF (Cognard type IV, Borden typeIII). The left external carotid angiogram showed left falcine artery and branches of MMA making arteriovenous shunt at the falcotentorial junction, draining to vein of Galen and making a large varix at the draining route (**A**: AP view and **B**: Lat view). Right vertebral angiogram showed branches of bilateral posterior cerebral artery, making arteriovenous shunt at the falcotentorial junction, draining to vein of Galen and making a large varix at the draining route (**C**: AP view and **D**: Lat view). First, we performed n-butyl-2-cyanoacrylate (NBCA) trans-arterial embolization (TAE) of left MMA petrosal branch (**E**: Lat view). However, post-TAE vertebral angiography showed left PCA peripheral flow reduction (**F**: AP view). Next, second NBCA TAE of right falcine artery was performed (**G**: AP view) 8 weeks after the first NBCA TAE. Postoperative DSA showed the angiographical disappearance of falco-tentorial dural AVF and recanalization of left PCA peripheral artery (**H**: AP view and **I**: Lat view)

**Fig. 3:**
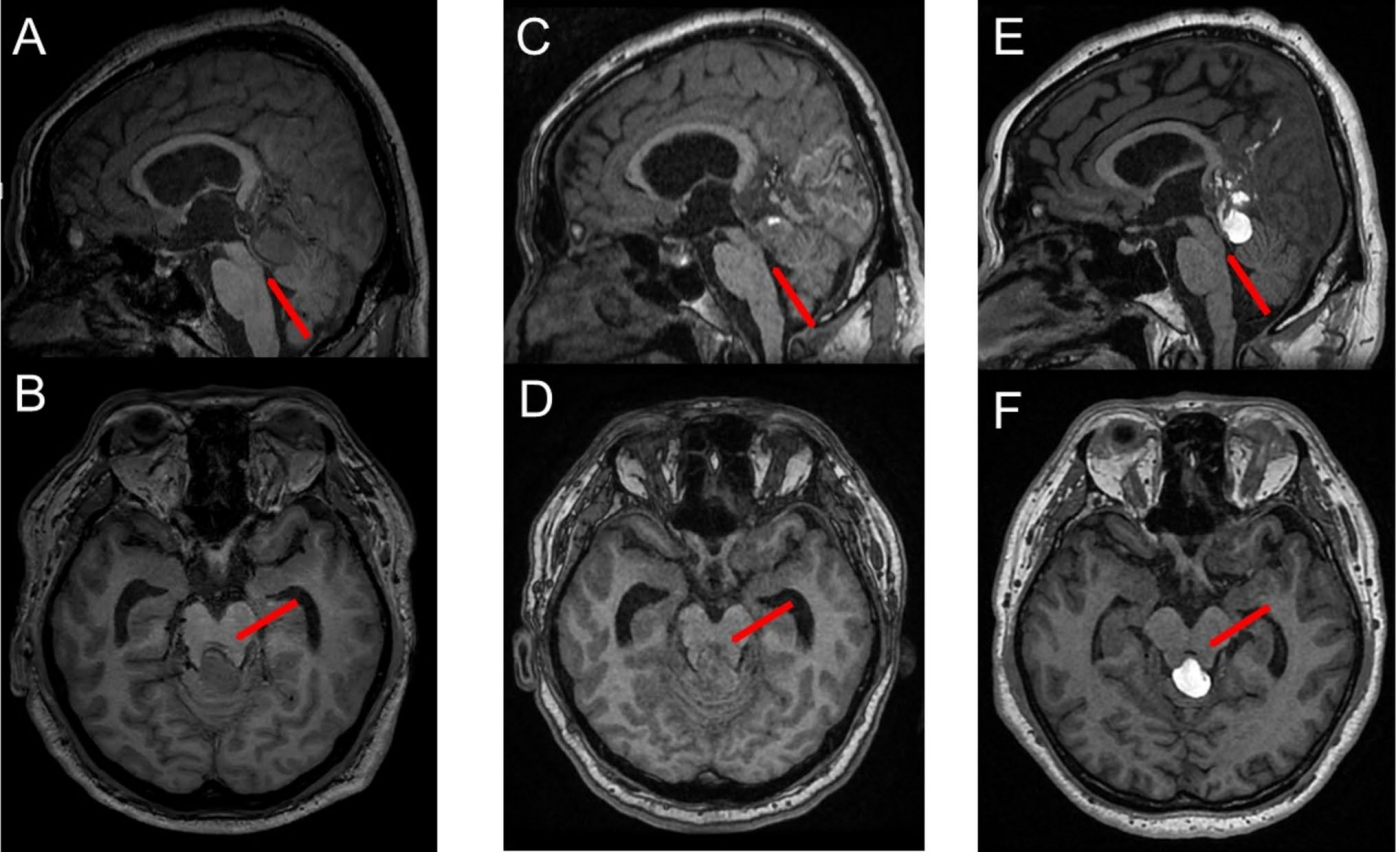
Initial brain magnetic resonance (MRI) showed a dilated varix of the dural AVF stenosed the aqueduct (red arrow) in the sagittal **A** and axial view **B**. Four days after the second TAE treatment of the dural AVF, disturbance of the consciousness worsened, hydrocephalus was worse on brain MRI **A**, showing the more dilated varix by the thrombus formation causing the occlusion of aqueduct (red arrow) in sagittal view **C** and axial view **D**. Six months after the TAE treatment, his brain MRI showed the shrinking of the dilated varix by the complete thrombus formation re-opening of the occluded aqueduct (red arrow) in sagittal view **E** and axial view **F**

A 54-year-old male presented with a six-month progressive history of gait disturbance, urinary incontinence, and headache. Initial head computed tomography (CT) showed several relatively high-density mass lesions in the quadrigeminal cistern causing occlusive hydrocephalus (Fig. 1A-C). Brain magnetic resonance imaging (MRI) showed dilated varix of the dural AVF, that stenosed the aqueduct, causing occlusive hydrocephalus (Fig. 3A, B). Moreover, digital subtraction angiography (DSA) confirmed a tentorial dural AVF (Cognard type IV, Borden type III) (Fig. 2A-I). The dural AVF was fed by several branches of the bilateral external carotid and posterior cerebral arteries, creating a large varix flowing into the vein of Galen.

**Fig. 4:**
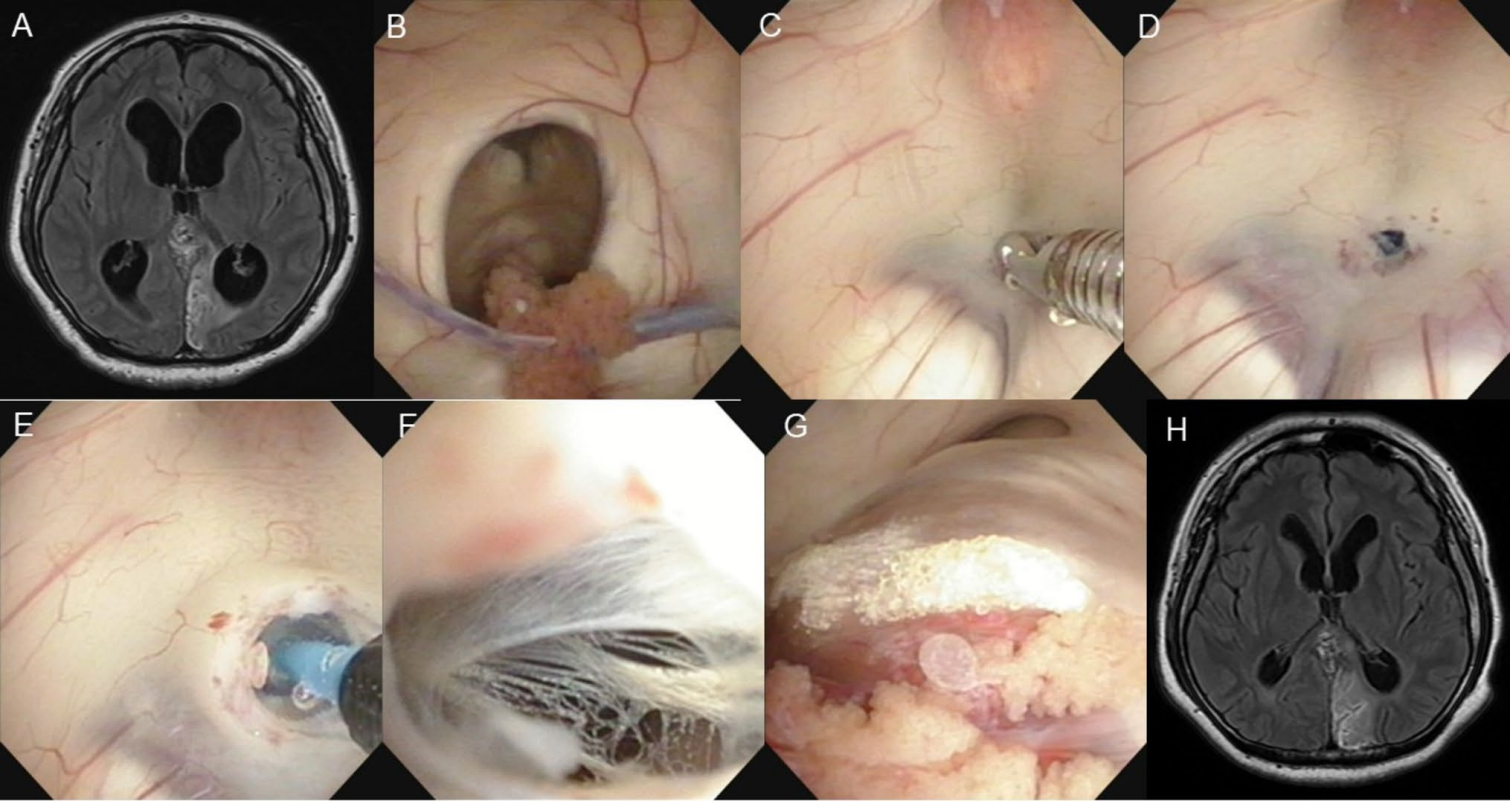
Four days after the second TAE treatment of the dural AVF, disturbance of the consciousness worsened, hydrocephalus was worse on his brain MRI **A**. Emergent endoscopic third ventriculostomy (ETV) was performed. Right foramen of Monro was dilated **B**. Puncture was made at the midpoint between the bilateral mammillary bodies and infundibular recess **C**, **D**. Puncture hole was dilated with balloon catheter **E**, making clearly visible to the quadrigeminal cistern **F**. Aqueductal occlusion due to the varix of the dural AVF was observed **G**. Post-ETV MRI showed the improvement of the hydrocephalus **H**

We first performed n-butyl-2-cyanoacrylate (NBCA) transarterial embolization (TAE) of the petrosal branch of the left middle meningeal artery (MMA) (Fig. 2e). Post-TAE vertebral angiography showed left PCA peripheral flow reduction (Fig. 2f). Post-TAE FLAIR image showed left occipital high signal intensity lesion and clinically mild right-side hemianopia was observed (Fig. 4a). This occipital lesion was thought to be cerebral ischemia caused by venous hypertension from the occlusion of venous drainage by the NBCA cast or temporally venous varix and drainage route compression to PCA trunk, causing PCA peripheral flow reduction.

Additional NBCA TAE of the right falcine artery was performed 8 weeks later (Fig. 2g). Postoperative DSA revealed angiographic disappearance of the falcotentorial dural AVF and recanalization of left PCA peripheral artery (Fig. 2h, i).

Four days after the second TAE treatment for the dural AVF, he presented with a disturbance of consciousness and his brain MRI revealed worsening hydrocephalus caused by the complete occlusion of the aqueduct by the dilated and thrombosed varix of the dural AVF (Figs. 3c, d and 4a). Emergent ETV was performed (Fig. 4b-g), resulting in improved consciousness and hydrocephalus on brain MRI (Fig. 4h). The patient was discharged with mild right-side hemianopia caused by left occipital lobe infarction.

At six-month follow-up, no recurrence of the dural AVF nor hydrocephalus was observed on brain MRI (Fig. 3e, f). The patient is currently doing well and performing normal activities of daily living (Table 1) [3, 8, 10, 12, 14, 15].

**Table 1:** Review of cases of hydrocephalus associated with dural AVF

Author	Year	Age	Gender	Symptoms	Location of dural AVF	Cognard Classification	Hydrocephalus	Treatment of dural AVF	Angiographical result	Addtional treatment for hydrocephalus	Follow up (month)
Monges et al.	2005	50 days	Female	Macrocrania	Torcular herophili	III	Occlusive	TAE	Complete resolution	VPS	36
Ernst et al.	2006	77 y.o	Female	Disturbance of conciousness	TS	II b	Occlusive	TAE (ONYX)	Complete resolution	None	6
Nakahara et al.	2011	76 y.o	Female	Disturbance of conciousness	TS-SS	III	Communicating	TVE	Complete resolution	None	Not applicable
Wen et al.	2016	76 y.o	Male	Progressive dementia, gait disturbance, urinary incontinence	TS, SSS	III	Communicating	TAE	Complete resolution	None	36
Zhang et al.	2019	55 y.o	Female	Headache, blurring vision	Falcotentorial	IV	Occlusive	TAE (ONYX)	Complete resolution	None	24
Ndandja et al.	2023	8 mo	Male	Macrocania	Torcular herophili	III	Occlusive	TAE	Complete resolution	None	Not applicable
Nejadhamzeeigilani et al.	2024	50 y.o	Male	Headache, confusion, unsteadiness	Falcotentorial	III	Occlusive	TAE (Squid-18)	Complete resolution	None	Not applicable
Present case	2024	54 y.o	Male	Gait disturbance, urine incontinence	Falcotentorial	IV	Occlusive	TAE (NBCA)	Complete resolution	ETV	3

*AVF* arterior venous fistulae, *ETV* endoscopic third ventriculostomy, *m.o* months, *NBCA* N-Butyl-2-cyanoacrylate, *SSS* superior sagittal sinus, *TAE* transarterial embolization, *TS* transverse sinus, *y.o* years-old, *VPS* ventricular peritoneal shunt

## Discussion

Dural AVFs are curable vascular lesions associated with arteriovenous shunts between the dural arteries and cerebral venous sinuses and/or cortical veins [1, 5]. They commonly affect individuals in their 50s and 60s [1, 5]. Headaches, vertigo, and tinnitus are the most prominent clinical symptoms [1, 5]. Severe cases, especially those with cerebral venous reflux from venous drainage, can lead to fatal intracranial hemorrhage [1, 5]. Additionally, increased venous pressure often results in an elevated intracranial pressure in some patients [1, 5].

Hydrocephalus is rarely observed in patients with dural AVF. We reviewed eight cases in the literature [3, 8–12, 14, 15], including our own, with patient ages ranging from 50 days to 77 years and equal sex distribution (men: 4 and women: 4). In children, dural AVFs were mostly observed in torcular herophili, whereas in adults, they were found in the transverse and/or sigmoid sinus (three cases) and the tentorial or falcotentorial region (three cases). The Cognard types were IIb (one case), III (five cases), and IV (two cases). Four occlusive and four communicating hydrocephalus types were found. Dural AVF treatment included TAE (7 cases) and transvenous embolization (TVE) (1 case), and in all cases, complete angiographic resolution was achieved. The treatment for hydrocephalus linked to dural AVF included a ventricular peritoneal (VP) shunt (1 case), ETV (1 case), and spontaneous resolution after TAE or TVE (6 cases). The follow-up term was 3 to 36 months.

Hydrocephalus accompanied by dural AVF is often due to cerebral aqueduct occlusion or stenosis by enlarged or thrombosed varix of dural AVF [3, 8, 10–12, 14, 15]. A previous case report has noted occlusive hydrocephalus resulting from posterior fossa venous congestion and secondary posterior fossa brain swelling [3]. Additionally, other cases have shown occlusive hydrocephalus caused by drainage into the deep venous system, resulting in dilation of the vein of Galen or secondary occurred large varix [6, 8, 11, 12, 15]. This pathology is similar to that of vein of Galen malformation (VGM) in infants, in the sense that obstructive hydrocephalus persists after TAE. This obstructive hydrocephalus results from obstruction of the aqueduct of Sylvius owing to the mass effect of aneurysmatic dilation of the VGM. In this situation, ETV is more effective than the VP shunt in improving the post-TAE obstructive hydrocephalus [4, 2, 7, 13].

Spontaneous resolution occurred in four cases after TAE and after TVE in eight cases of occlusive hydrocephalus linked to dural AVF. To our knowledge, our case is the first case report where ETV could effectively resolve occlusive hydrocephalus. Furthermore, occlusive hydrocephalus was caused by aqueduct obstruction from a large varix, which worsened due to acute thrombosis of the varix and drainage route following TAE for dural AVF treatment. The presence of a large varix near the midbrain, in our case, could have been a risk factor for worsening hydrocephalus after TAE. The dural AVFs were successfully treated with TAE using NBCA; however, acute thrombosis of the drainage route, particularly the varix acute dilation by the thrombosis formation, worsened the occlusive hydrocephalus. Interestingly, as in our case, the thrombosed varix was dilated at the acute phase after the TAE treatment, which gradually shrunk later. Treatment options for occlusive hydrocephalus include ETV and a VP shunt. We recommend ETV as the first choice, because it achieves a “VP shunt free” state, and occlusive hydrocephalus can be resolved as the thrombosed dilated varix shrinks. In the present case, the patient was discharged without neurological deficits caused by occlusive hydrocephalus or recurrence of occlusive hydrocephalus post-ETV state.

## Conclusion

Occlusive hydrocephalus associated with dural AVF is extremely rare. In most cases, resolution of hydrocephalus after dural AVF treatment is achieved; however, as in this case, if additional treatment for the hydrocephalus is needed after dural AVF treatment, ETV may be considered a treatment option for the hydrocephalus.

## Data Availability

No datasets were generated or analysed during the current study.
